# Examining changes in recreation participation on the Monongahela National Forest over five years following the global financial crisis: An activity-based segmentation approach

**DOI:** 10.1016/j.heliyon.2021.e07266

**Published:** 2021-06-10

**Authors:** Chad D. Pierskalla, Connor B. Akers, Jinyang Deng, David A. Smaldone

**Affiliations:** West Virginia University, Recreation, Parks and Tourism Resources, PO Box 6125, Morgantown, WV 26506, USA

**Keywords:** Financial crisis, Market segmentation, Activity-based segmentation, Monongahela National Forest, NVUM

## Abstract

Research on recreational use market segments and trends are essential for determining how tourism businesses can better meet the needs of their customers and find new target markets especially during challenging times. The purpose of this study was to determine how recreation participation has changed over five years on the Monongahela National Forest following the world financial crisis of 2008–2009. Data used for this research were collected with the National Visitor Use Monitoring (NVUM) surveys for fiscal years 2009 and 2014. NVUM surveys are on-site interviews conducted at the end of a visit. The surveys produce descriptive information about visitors. NVUM uses a stratified random sampling methodology to collect data for each use level (e.g., low, medium, high, or very high) and site type (e.g., Day Use Developed Sites, Overnight Use Developed Sites, General Forest Areas, and Wilderness Sites). Data were collected during a 12-month period and a total of 1,851 interviews were completed. Two step cluster analysis of 18 activity participation variables (binary data) was conducted with 5 clusters specified. The clusters include Relaxing in Nature Package, Backpacking and Hiking Package, Viewing Nature and Hunting Package, Picnicking Package, and Purely Fishing, and they were compared with an activity package typology. Changes in demographics, trip spending, nights away from home, and overall satisfaction were examined for each market segment. Potential market winners and losers during the financial crisis were identified helping tourism providers develop more efficient strategies.

## 1 Introduction

The recent outbreak of Coronavirus (COVID-19) and its impact on the global economy is a reminder that tourism and crisis is a never-ending story, and when it happens, the profession should learn from it. Previous to that outbreak, travel and tourism was hit hard by the 2008–2009 financial crisis which was the deepest recession since the Great Depression (Meng et al., 2010; WTTC, 2010).

The deep economic crisis severely affected employees' work and personal lives. Hochwarter (2009) showed that 70 percent of both men and women in his study confirmed that the recession increased stress levels. More than 70 percent also admitted making spending changes, including limiting or eliminating the purchase of non-essential items. Mucci et al. (2016) implemented a systematic review of the principal studies that examined the impact of the crisis on the health of workers. A total of 19 articles were reviewed. All of the studies showed that the crisis was a stressor that impacted workers' mental health. There were increases in mood disorders, dysthymia, anxiety disorders, somatoform disorders, panic attacks, and abuse of alcohol.

The impact of the macro-economic event on tourism was also felt all around the world because the purchasing power of people significantly influences their decisions to travel (Mihalič et al., 2013). Rittchainuwat et al. (2014) found that novelty, culture and safe and short-distance destinations would motivate tourists to travel during the financial crises because it saves time, effort, and travel costs. Within Europe, a contraction of travel in time and space was reported. For example, “the average German's current approach…can be described in the following way: closer, shorter, cheaper” (Reinhardt, 2011, p. 27).

Much of the literature on the global economic downturn implies that the effects have been ubiquitous and the same negative patterns were experienced universally. In fact, the popular media was well supplied with stories about the challenges faced by the travel and tourism industry during the financial crisis (Coles, 2013), but little research has been published at smaller scales. For example, there is a lack of literature specifically on crisis-impacted market segments. There have been winners and losers within tourism (Coles, 2013), and perhaps they can be more easily identified within smaller markets or at smaller scales like a national forest. When market segments are better understood, more efficient strategies can be developed to meet the needs of target markets, attract new markets, promote product development, better allocate marketing resources, manage visitor conflict, or increase customer volume in off-peak periods (Choi and Tsang, 2000; Mumuni and Mansour, 2014).

The purpose of this study was to determine how recreation participation has changed over five years on the Monongahela National Forest following the world financial crisis in fiscal year 2009. Data collection started on October 1, 2008 just two weeks after Lehman Brothers filed for bankruptcy which was near the beginning of the crisis. The study objectives include:

(1) identify and describe the potential market winners and losers during the financial crisis and
(2) examine the variety of outdoor recreation activities within each market segment by comparing them with an activity package typology that is presented in the literature review.

## 2 Literature review

### 2.1 Market segmentation

Market segmentation is at the heart of modern marketing (Schneider et al., 2006), and is one of the most important management tools of the recreation and tourism planner (Schroeder, 1987). Tourism segmentation involves grouping tourists into homogenous categories based on the similarity with one or more variables including travel motivation, benefits sought, travel mode, expenditure amounts and patterns, and preferences for tourism activities, among others (Mumuni and Mansour, 2014). Statistical techniques such as factor and/or cluster analysis are used to identify the distinct segments when using *a posteriori* approach (versus identifying clusters *a priori*). The resulting segments are then profiled based on similarity with other variables such as sociodemographics.

### 2.2 Activity-based segmentation

Activities are an effective segmentation base (Pesonen and Tuohino, 2017) and are the key product in adventure travel (Schneider et al., 2006). Unlike motivations, an advantage of activity-based segmentation is that it is stable across different national and cultural contexts (Mumuni and Mansour, 2014). Activities also serve as a link between travel motivation and destination choice (Cooper et al., 2005). Beritelli and Boksberger (2005) link tourist activities and motivations further supporting the important role activities play in destination marketing. In addition, grouping activities into packages through segmentation can provide more convenience for travelers and can increase the desire for specialized activities (Hsieh et al., 1992).

Classification of leisure activities have been important to leisure research because science is based on the groupings of unique events or objects into similarity classes (Williams, 1988). McCool (1978) defined an activity package as a set of activities in which the visiting group engages at a recreation area. One of the first and best examples of a study on activity type, especially as it relates to forest recreation packages, was conducted by Hendee et al. (1971). Respondents in their study were asked to identify their six most preferred activities from a list of twenty-six conceivable recreation activities. They proposed five conceptually linked activity packages based on "perceived similarities in the underlying meanings of the activities to the participant" (McCool, 1978, p. 166). The typology is unique because it is based on the study of recreationists visiting both car campgrounds and wilderness in national parks and national forests, therefore it is most suitable for this study of national forest visitors. In addition, the study was among three studies that Manning (2011) selected to illustrate the genre of research on activity type. McCool (1978) added additional examples of activities to the five activity packages which are summarized below (somewhat abbreviated):

• *Appreciative-Symbolic*: Activities directed toward appreciation of features of the natural environment. The recreationist's focus is on appreciation of material items in the environment rather than on their extraction in the form of "trophies." Examples of activities include seeing natural scenery on foot or horseback, hiking, and photography.
• *Extractive-Symbolic*: Activities characterized by the quest for trophies extracted from the natural environment. Examples of activities include fishing and hunting.
• *Passive-Free Play*: Activities requiring little effort and not confined to the forest environment. Examples of activities include relaxing, driving for pleasure, quiet boating or canoeing, and picnicking.
• *Sociable-Learning*: Social and learning activities such as nature study, hearing nature talks, visiting exhibits, visiting historic sites, and visiting with other people.
• *Active-Expressive*: Activities not requiring use of a forest setting and which, in fact, sometimes interfere with other activities at the same site. Examples of activities include swimming, jogging, bicycling, golf, and organized games such as playing softball, football, and horseshoes.

This study uses market segmentation, particularly activity-based segmentation, to create activity packages that can be compared with Hendee et al.’s (1971) typology. Few studies, if any, have directly compared their activity groupings with a typology. This is a gap in the literature that is addressed by this paper.

Activity-based segmentation has proved useful for many different types of tourist and has a wide array of support. Schneider et al. (2006) cited a range of studies that use activity-based segmentation including segmentation for culture tourists (McKercher et al., 2002), nature tourists (Lang and O'Leary, 1997), ecotourists (Wight, 1996), adventure tourists (Sung et al., 1997), and visiting friends and relatives tourist market (Moscardo et al., 2000). The authors added studies that have been published since 2006 (Table 1). Recent literature represents markets from the US (Schneider et al., 2006), Canada (Choi et al., 2011), South Africa (Boekstein and Spencer, 2013), Saudi Arabia (Mumuni and Mansour, 2014), Norway (Tkaczynski et al., 2015), Croatia (Barić et al., 2016), Finland (Pesonen and Tuohino, 2017), and Portugal (Eusébio et al., 2017). Despite recent data that are available (like the data used in this study), none of this literature examined visitor activities on national forest land in the US. Together, the number of segments that were identified in these activity-based segmentation studies ranged from two to eight with a median of four segments.

**Table 1:** Activity segmentation literature updated from Schneider et al. (2006).

Author	Date	Sample	Name of Segments
Schneider, Vogt, & Smith	2006	US adventure travel market	• Soft nature • Cerebral Pursuit • Question marks • Expedition discovery
Choi, Murraya, & Kwana	2011	Canadian domestic pleasure travelers to New Brunswick	• Outdoor lovers • Active explorers • Cultural shoppers
Boekstein & Spencer	2013	Thermal spring resorts in South Africa	• Passive families • Passive relaxers • Active outdoors • Active families
Mumuni & Mansour	2014	Saudi Arabia's outbound leisure tourism market	• Conservatives • Variety seekers • Fun seekers
Tkaczynski Rundle-Thiele, & Prebensen	2015	Norway's nature-based tourists based on summer, winter, and year-round activity preferences	• Experiencing the midnight sun (summer) • Experiencing the northern lights (winter) • Experiencing frords (year-round)
Barić, Anić, & Bedoya	2016	Croatia's protected area visitors	• Activists • Passivists
Pesonen & Tuohino	2017	Finland's rural well-being tourists	• Sporties • Wellbing enthusiasts • Spa goers
Eusébio, Carneiro, Kastenholz, Figueiredo, & da Silva	2017	Portugal's domestic rural tourism market	• The active visitors • The passive nature observers • The inactives • The summer family vacationers

## 3 Method

The study was conducted on the Monongahela National Forest which is located in the Allegheny Mountains in eastern West Virginia. The forest consists of over 921,000 acres of federally owned land. Tourism is an increasingly important part of the West Virginia economy given the volatility of the coal mining industry.

The authors used data that they helped collect for the National Visitor Use Monitoring (NVUM) program of the US Forest Service. The objective of the program is to estimate the volume of recreation visitation to all national forests and grasslands in the US and to analyze visitation with respect to activity participation, demographics, trip spending, travel distance, etc. The revised method was first applied in 2000. The 12-month studies are conducted on a 5-year sampling cycle. This study's methodology is supported by other studies that also used NVUM data such as Askew et al. (2017).

### 3.1 Data collection

Five categories of site types are used to help define the sampling frame and include Day Use Developed Sites, Overnight Use Developed Sites, Designated Wilderness Areas, General Forest Areas, and View Corridors. On-site exit interviews were conducted at those sites on days selected using a stratified random sampling design. Stratified sampling was based on site type and exiting use level (i.e., low, medium, high, and very high). The interview period was 6 h each day with a morning or afternoon start time randomly determined. One person with the most recent birthday and 16 years of age or older was selected from each group to participate in the study. The sampling of visitors screens whether a person has recreated on the site/forest and the interview collects information immediately after a visit. Interviews took place from October 1 to September 30 during fiscal years 2009 and 2014. Data were collected by West Virginia University and the Appalachian Forest Heritage staff.

### 3.2 Data analysis

Data were analyzed using IBM SPSS Statistics Version 25. Two-step cluster analysis of 18 activity participation variables (binary "yes" or "no" data) were conducted with 3–5 clusters examined to find the most logical solution for the entire data set. Two-way analysis of variance (ANOVA) was used to examine two categorical independent variables (i.e., fiscal year of study and cluster membership) and one continuous dependent variable (i.e., travel distance, trip spending, nights away from home, and overall satisfaction). The authors compared the association between activity participation, primary activity, cluster membership, and demographics with fiscal year using Chi-square tests.

## 4 Results

### 4.1 Segmentation procedure

The sample size was 823 visitors in fiscal year 2009 and 1,028 in fiscal year 2014. A two-step cluster analysis was used to segment visitors to the Monongahela National Forest using all of the data collected during both years. That is, visitors who participated in similar activities were grouped together. Based on criteria provided by Weinstein (1987), a five-cluster solution was selected. The criteria include homogeneity within the segment, heterogeneity between segments, sizable population, and meaningful segment data (e.g., segment data that are most practical and useable). Each cluster was given a name based on the package of activities and the primary activities reflected in the cluster.

### 4.2 Relaxing in Nature Package

Cluster 1 (n = 577) represented 31.2 percent of the total sample in this study and was labeled Relaxing in Nature Package (Table 2). Viewing nature had the highest percentage of visitors (95%) within the cluster. The percentages associated with activity participation that were among the highest across clusters include hiking (82%), relaxing (81.8%), viewing wildlife (80.8%), driving for pleasure (67.8%), nature centers (50.8%), history (35.4%), nature study (25.6%), and resorts (9.4%). Visitors were also asked to select their one primary activity among the list of activities. The most common primary activities complement activity participation (Table 3).

**Table 2:** Activity participation by cluster membership.

Activity	Cluster 1 (n = 577)	Cluster 2 (n = 296)	Cluster 3 (n = 483)	Cluster 4 (n = 252)	Cluster 5 (n = 242)	χ^2^
Backpacking	60 (10.4%)	**148 (50%)**	4 (.8%)	13 (5.2%)	1 (.4%)	497.381∗∗∗
Biking	51 (8.8%)	0 (0%)	5 (1%)	**42 (16.7%)**	10 (4.1%)	103.109∗∗∗
Developed Camping	165 (28.6%)	24 (8.1%)	37 (7.7%)	**84 (33.3%)**	70 (28.9%)	135.76∗∗∗
Driving for Pleasure	**391 (67.8%)**	31 (10.5%)	76 (15.7%)	70 (27.8%)	17 (7%)	535.63∗∗∗
Fishing	104 (18%)	28 (9.5%)	0 (0%)	145 (57.5%)	**241 (99.6%)**	991.025∗∗∗
Gathering Mushrooms etc.	98 (17%)	82 (27.7%)	12 (2.5%)	39 (15.5%)	6 (2.5%)	138.607∗∗∗
Relaxing	**472 (81.8%)**	161 (54.4%)	63 (13%)	171 (67.9%)	24 (9.9%)	685.674∗∗∗
Hiking	**473 (82%)**	**231 (78%)**	122 (25.3%)	**178 (70.6%)**	0 (0%)	724.046∗∗∗
History	**204 (35.4%)**	2 (.7%)	6 (1.2%)	3 (1.2%)	0 (0%)	460.15∗∗∗
Hunting	6 (1%)	4 (1.4%)	**34 (7%)**	6 (2.4%)	3 (1.2%)	42.098∗∗∗
Nature Centers	**293 (50.8%)**	0 (0%)	33 (6.3%)	6 (2.4%)	1 (.4%)	618.203∗∗∗
Nature Study	**148 (25.6%)**	13 (4.4%)	3 (.6%)	7 (2.8%)	0 (0%)	273.457∗∗∗
Non-Motorized Water	22 (3.8%)	0 (0%)	1 (.2%)	8 (3.2%)	0 (0%)	34.924∗∗∗
Picnicking	220 (38.1%)	6 (2%)	5 (1%)	**128 (50.8%)**	0 (0%)	507.72∗∗∗
Primitive Camping	66 (11.4%)	13 (4.4%)	0 (0%)	33 (13.1%)	21 (8.7%)	70.477∗∗∗
Resorts	**54 (9.4%)**	9 (3%)	7 (1.4%)	0 (0%)	2 (.8%)	70.666∗∗∗
Viewing Nature	**548 (95%)**	213 (72%)	**181 (37.5%)**	39 (15.5%)	0 (0%)	912.946∗∗∗
Viewing Wildlife	**466 (80.8%)**	**193 (65.2%)**	2 (.4%)	41 (16.3%)	0 (0%)	1029.826∗∗∗

Notes. ∗*p* < 0.05; ∗∗*p* < 0.01; ∗∗∗*p* < 0.001.

Cluster 1: Relaxing in Nature Package; Cluster 2: Backpacking and Hiking Package; Cluster 3: Viewing Nature and Hunting Package; Cluster 4: Picnicking Package; Cluster 5: Purely Fishing.

**Table 3:** Primary activity participation by cluster membership.

Activity	Cluster 1 (n = 577)	Cluster 2 (n = 296)	Cluster 3 (n = 483)	Cluster 4 (n = 252)	Cluster 5 (n = 242)	χ^2^
Backpacking	20 (3.5%)	86 (29.1%)	4 (.8%)	7 (2.8%)	0 (0%)	312.403∗∗∗
Biking	6 (1%)	0 (0%)	3 (.6%)	6 (2.4%)	0 (0%)	12.715∗
Developed Camping	28 (4.9%)	5 (1.7%)	21 (4.3%)	29 (11.5%)	23 (9.5%)	33.435∗∗∗
Driving for Pleasure	43 (7.5%)	5 (1.7%)	40 (8.3%)	8 (3.2%)	1 (.4%)	35.701∗∗∗
Fishing	43 (7.5%)	19 (6.4%)	0 (0%)	82 (32.5%)	213 (88%)	961.191∗∗∗
Gathering mushrooms, etc.	1 (.2%)	3 (1%)	11 (2.3%)	3 (1.2%)	0 (0%)	14.866∗∗∗
Relaxing	64 (11.1%)	11 (3.7%)	17 (3.5%)	26 (10.3%)	3 (1.2%)	47.631∗∗∗
Hiking	146 (25.3%)	107 (36.1%)	69 (14.3%)	21 (8.3%)	0 (0%)	156.492∗∗∗
History	7 (1.2%)	1 (.3%)	2 (.4%)	0 (0%)	0 (0%)	7.91
Hunting	2 (.3%)	2 (.7%)	32 (6.6%)	4 (1.6%)	3 (1.2%)	54.695∗∗∗
Nature Centers	1 (.2%)	0 (0%)	14 (2.9%)	0 (0%)	0 (0%)	31.786∗∗∗
Nature Study	5 (.9%)	0 (0%)	1 (.2%)	0 (0%)	0 (0%)	8.024
Non-Motorized Water	6 (1%)	0 (0%)	5 (1%)	3 (1.2%)	0 (0%)	5.848
Picnicking	23 (4%)	0 (0%)	2 (.4%)	35 (13.9%)	0 (0%)	122.377∗∗∗
Primitive Camping	9 (1.6%)	1 (.3%)	0 (0%)	12 (4.8%)	3 (1.2%)	31.096∗∗∗
Resorts	2 (.3%)	0 (0%)	0 (0%)	0 (0%)	0 (0%)	4.417
Viewing Nature	91 (15.8%)	19 (6.4%)	122 (25.3%)	2 (.8%)	0 (0%)	152.1∗∗∗
Viewing Wildlife	24 (4.2%)	10 (3.4%)	2 (.4%)	0 (0%)	0 (0%)	33.743∗∗∗

Notes. ∗*p* < 0.05; ∗∗*p* < 0.01; ∗∗∗*p* < 0.001.

Cluster 1: Relaxing in Nature Package; Cluster 2: Backpacking and Hiking Package; Cluster 3: Viewing Nature and Hunting Package; Cluster 4: Picnicking Package; Cluster 5: Purely Fishing.

### 4.3 Backpacking and Hiking Package

Cluster 2 (n = 296) consisted of 16 percent of the total sample and was labeled Backpacking and Hiking Package (Table 2). Most respondents participated in hiking (78%). Other activities include viewing wildlife (65.2%) and backpacking (50%), and both reflect the highest percentages across clusters. The selection of a primary activity complements these findings (Table 3).

### 4.4 Viewing Nature and Hunting Package

Cluster 3 (n = 483) represented 26.1 percent of the sample and was labeled Viewing Nature and Hunting Package. Compared to other clusters, hunting participation (7%) was highest for this cluster (Table 2). Viewing nature was also selected as the primary activity (25.3%) more often than the other four clusters (Table 3).

### 4.5 Picnicking Package

Cluster 4 (n = 252) represented 13.6 percent of the sample and was labeled Picnicking Package. Participation in picnicking (50.8%) along with picnicking as a primary activity (13.9%) was highest for this cluster (Tables 2 and 3). Hiking participation (70.6%), developed camping (33.3%), and biking (16.7%) were also among the highest percentages across clusters.

### 4.6 Purely Fishing

Cluster 5 (n = 242) represented 13.1 percent of the sample and was labeled Purely Fishing. This cluster represents visitors who exclusively participate in fishing (99.6%) and selected fishing as the primary activity (88%) (Tables 2 and 3).

### 4.7 Fiscal year differences in cluster membership

There were significant winners and losers among the different market segments following the global financial crisis (χ^2^ = 89.4, *p* < .001) (Table 4). Visitors participating in the Relaxing in Nature Package were 13.9 percent higher in fiscal year 2009. Participation in Backpacking and Hiking (10.6% lower) and Purely Fishing (8.4% lower) were among the losing market segments during the financial crisis.

**Table 4:** Cluster membership by fiscal year (FY).

Cluster	FY09	FY14	χ^2^
1	**320 (38.9%)**	257 (25%)	89.431∗∗∗
2	83 (10.1%)	**213 (20.7%)**	
3	236 (28.7%)	247 (24.1%)	
4	115 (14%)	137 (13.3%)	
5	69 (8.4%)	**173 (16.8%)**	

Notes. ∗*p* < 0.05; ∗∗*p* < 0.01; ∗∗∗*p* < 0.001.

Cluster 1: Relaxing in Nature Package; Cluster 2: Backpacking and Hiking Package; Cluster 3: Viewing Nature and Hunting Package; Cluster 4: Picnicking Package; Cluster 5: Purely Fishing.

### 4.8 Fiscal year differences in demographics

Demographics including gender, age, and household income were compared by fiscal year for each market segment (Table 5). The only significant difference (χ^2^ = 13.59, *p* < .05) among fiscal years was age for the Purely Fishing cluster. There were fewer cluster members for age groups 50–59, 60–69, and 70+ during the financial crisis. That is, older anglers were less likely to participate in fishing during fiscal year 2009.

**Table 5:** Visitor demographics by fiscal year (FY).

		FY09	FY14	χ^2^
Cluster 1	Male	207 (64.9%)	148 (57.8%)	3.012
	Female	112 (35.1%)	108 (42.2%)	

	Under 16	2 (0.6%)	0 (0%)	13.798
	16–19	5 (1.6%)	0 (0%)	
	20–29	49 (15.4%)	35 (13.7%)	
	30–39	56 (17.6%)	49 (19.1%)	
	40–49	59 (18.5%)	50 (19.5%)	
	50–59	86 (27.0%)	54 (21.1%)	
	60–69	47 (14.7%)	43 (16.8%)	
	70+	15 (4.7%)	25 (9.8%)	

	Under $25,000	12 (12.2%)	6 (9.8%)	11.418
	$25,000 - $49,000	28 (28.6%)	7 (11.5%)	
	$50,000 - $74,000	23 (23.5%)	17 (27.9%)	
	$75,000 - $99,000	15 (15.3%)	12 (19.7%)	
	$100,000 - $149,999	16 (16.3%)	13 (21.3%)	
	$150,000 or over	4 (4.1%)	6 (9.8%)	
Cluster 2	Male	65 (78.3%)	149 (70.6%)	1.782
	Female	18 (21.7%)	62 (29.4%)	

	Under 16	1 (1.2%)	0 (0%)	8.207
	16–19	1 (1.2%)	0 (0%)	
	20–29	12 (14.5%)	35 (13.6%)	
	30–39	16 (19.3%)	49 (19.1%)	
	40–49	18 (21.7%)	50 (19.5%)	
	50–59	18 (21.7%)	54 (21.0%)	
	60–69	14 (16.9%)	43 (16.7%)	
	70+	3 (3.6%)	25 (9.7%)	

	Under $25,000	2 (8.7%)	6 (10.0%)	0.791
	$25,000 - $49,000	6 (26.1%)	17 (28.3%)	
	$50,000 - $74,000	8 (34.8%)	16 (26.7%)	
	$75,000 - $99,000	3 (13.0%)	7 (11.7%)	
	$100,000 - $149,999	2 (8.7%)	8 (13.3%)	
	$150,000 or over	2 (8.7%)	6 (10.0%)	
Cluster 3	Male	120 (65.9%)	141 (71.6%)	1.403
	Female	62 (34.1%)	56 (28.4%)	

	Under 16	5 (2.8%)	1 (0.5%)	9.523
	16–19	3 (1.7%)	7 (3.5%)	
	20–29	28 (15.5%)	34 (17.2%)	
	30–39	38 (21%)	27 (13.6%)	
	40–49	33 (18.2%)	31 (15.7%)	
	50–59	32 (17.7%)	44 (22.2%)	
	60–69	27 (14.9%)	33 (16.7%)	
	70+	15 (8.3%)	21 (10.6%)	

	Under $25,000	3 (5.6%)	7 (14.3%)	5.943
	$25,000 - $49,000	13 (24.1%)	13 (26.5%)	
	$50,000 - $74,000	15 (27.8%)	12 (24.5%)	
	dollar;75,000 - $99,000	9 (16.7%)	3 (6.1%)	
	$100,000 - $149,999	5 (9.3%)	7 (14.3%)	
	$150,000 or over	9 (16.7%)	7 (14.3%)	
Cluster 4	Male	77 (67.5%)	96 (70.6%)	0.27
	Female	37 (32.5%)	40 (29.4%)	

	Under 16	2 (1.7%)	0 (0%)	6.83
	16–19	8 (7.0%)	3 (2.2%)	
	20–29	13 (11.4%)	15 (11.0%)	
	30–39	22 (19.3%)	31 (22.8%)	
	40–49	24 (21.1%)	31 (22.8%)	
	50–59	17 (14.9%)	25 (18.4%)	
	60–69	23 (20.2%)	24 (17.6%)	
	70+	5 (4.4%)	7 (5.1%)	

	Under $25,000	5 (16.1%)	1 (3.7%)	6.842
	$25,000 - $49,000	8 (25.8%)	5 (18.5%)	
	$50,000 - $74,000	9 (29.0%)	11 (40.7%)	
	$75,000 - $99,000	6 (19.4%)	6 (22.2%)	
	$100,000 - $149,999	2 (6.5%)	4 (14.8%)	
	$150,000 or over	1 (3.2%)	0 (0%)	
Cluster 5	Male	63 (91.3%)	155 (91.2%)	0.001
	Female	6 (8.7%)	15 (8.8%)	

	Under 16	0 (0%)	0 (0%)	13.59∗
	16–19	2 (2.9%)	1 (0.6%)	
	20–29	9 (13.0%)	12 (7.0%)	
	30–39	7 (10.1%)	20 (11.7%)	
	40–49	19 (27.5%)	28 (16.4%)	
	50–59	15 (21.7%)	46 (26.9%)	
	60–69	16 (23.2%)	45 (26.3%)	
	70+	1 (1.4%)	19 (11.1%)	

	Under $25,000	3 (16.7%)	2 (6.3%)	8.513
	$25,000 - $49,000	6 (33.3%)	14 (43.8%)	
	$50,000 - $74,000	3 (16.7%)	11 (34.4%)	
	$75,000 - $99,000	4 (22.2%)	3 (9.4%)	
	$100,000 - $149,999	1 (5.6%)	2 (6.2%)	
	$150,000 or over	1 (5.6%)	0 (0%)	

Notes. ∗*p* < 0.05; ∗∗*p* < 0.01; ∗∗∗*p* < 0.001.

Cluster 1: Relaxing in Nature Package; Cluster 2: Backpacking and Hiking Package; Cluster 3: Viewing Nature and Hunting Package; Cluster 4: Picnicking Package; Cluster 5: Purely Fishing.

### 4.9 Fiscal year and cluster membership differences in travel distance

Two-way analysis of variance was used to examine differences in travel distance by fiscal year, cluster membership, and the interaction between the two variables (Table 6), and cluster membership (*p* < .001) and interaction effects were significant (*p* < .05). Tukey's post hoc comparisons (*p* < .05) were also examined. Visitors included in cluster 1 traveled farther than clusters 4 and 5 to get to the Monongahela National Forest, especially in fiscal year 2009 (Table 7). Cluster 2 and 3 visitors traveled farther than cluster 5 visitors.

**Table 6:** ANOVA summary table for travel distance.

Source	*df*	*MS*	*F*	*p*
FY	1	748553.17	2.59	.108
Cluster	4	2486618.83	8.59	<.001
FY × Cluster	4	735841.05	2.54	.038

Note: MS = Mean Squares.

**Table 7:** Descriptive statistics for travel distance.

FY	Cluster	Mean (Miles)	Std Deviation	N
2009	1	344.73	632.32	320
	2	426.24	1110.86	83
	3	256.21	545.38	182
	4	130.51	140.97	115
	5	92.97	79.44	69
2014	1	292.71	577.24	256
	2	192.16	202.90	212
	3	242.33	757.42	198
	4	165.40	221.62	136
	5	127.97	130.41	171

Note: Cluster 1: Relaxing in Nature Package; Cluster 2: Backpacking and Hiking Package; Cluster 3: Viewing Nature and Hunting Package; Cluster 4: Picnicking Package; Cluster 5: Purely Fishing.

### 4.10 Fiscal year and cluster membership differences in trip spending

Two-way analysis of variance was used to examine the effect of fiscal year and cluster membership on total trip spending (Table 8). Trip spending was significantly dependent on cluster membership (*p* < .01) but not fiscal year. Specifically, Tukey's post hoc tests indicated that cluster 3 visitors spent more than clusters 2, 4, and 5 (*p* < .05) during their trip regardless of fiscal year (Table 9).

**Table 8:** ANOVA summary table for trip spending.

Source	*df*	*MS*	*F*	*p*
FY	1	132300.35	0.28	.595
Cluster	4	2010127.62	4.30	.002
FY × Cluster	4	874794.33	1.87	.114

Note: MS = Mean squares.

**Table 9:** Descriptive statistics for trip spending.

FY	Cluster	Mean	Std Deviation	N
2009	1	412.51	482.16	97
	2	313.80	405.53	25
	3	724.46	1373.95	54
	4	193.52	162.77	29
	5	171.83	248.58	18
2014	1	493.37	674.50	67
	2	247.42	610.18	69
	3	386.63	737.64	63
	4	229.38	202.52	32
	5	274.33	463.87	43

Note: Cluster 1: Relaxing in Nature Package; Cluster 2: Backpacking and Hiking Package; Cluster 3: Viewing Nature and Hunting Package; Cluster 4: Picnicking Package; Cluster 5: Purely Fishing.

### 4.11 Fiscal year and cluster membership differences in nights away from home during trip

Respondents were asked to report the number of nights they would be away from home during their trip. Nights away from home was significantly dependent on cluster membership (*p* < .01), and it approached significance (*p* = .07) with fiscal year (Table 10). Mean scores were greater in fiscal year 2009 for all clusters except cluster 5. Tukey's post hoc tests indicated that cluster 1 visitors spent more nights away from home than clusters 2 and 5 (*p* < .05) (Table 11).

**Table 10:** ANOVA summary table for nights away from home.

Source	*df*	*MS*	*F*	*p*
FY	1	109.29	3.26	.071
Cluster	4	132.40	3.95	.003
FY × Cluster	4	43.12	1.29	.273

Note: MS = Mean squares.

**Table 11:** Descriptive statistics for nights away from home.

FY	Cluster	Mean	Std Deviation	N
2009	1	4.18	7.49	318
	2	2.90	3.41	83
	3	3.68	10.83	182
	4	2.88	3.71	115
	5	1.93	2.62	69
2014	1	3.34	4.91	255
	2	2.04	2.30	212
	3	2.13	3.69	198
	4	2.83	4.78	136
	5	2.45	3.55	169

Note: Cluster 1: Relaxing in Nature Package; Cluster 2: Backpacking and Hiking Package; Cluster 3: Viewing Nature and Hunting Package; Cluster 4: Picnicking Package; Cluster 5: Purely Fishing.

### 4.12 Fiscal year and cluster membership differences in overall satisfaction

Respondents were also asked to rate their overall trip experience on a 5-point scale ranging from 1 (very dissatisfied) to 5 (very satisfied). Overall trip satisfaction was significantly dependent on fiscal year (*p* < .001), cluster membership (*p* < .001), and the interaction effect (*p* < .05) (Table 12). Cluster 1 was significantly greater than clusters 3, 4, and 5 (*p* < .05). Cluster 2 was significantly greater than 3 and 5 (p < .05) (Table 13). Means scores were also higher among clusters during fiscal year 2014 (*p* < .05).

**Table 12:** ANOVA summary table for overall satisfaction.

Source	*df*	*MS*	*F*	*p*
FY	1	6.07	15.64	<.001
Cluster	4	7.86	20.27	<.001
FY × Cluster	4	11.11	2.87	.022

Note: MS = Mean squares.

**Table 13:** Descriptive statistics for overall satisfaction.

FY	Cluster	Mean	Std Deviation	N
2009	1	4.78	0.58	320
	2	4.88	0.33	83
	3	4.60	0.79	182
	4	4.71	0.65	115
	5	4.23	1.13	69
2014	1	4.87	0.42	256
	2	4.87	0.45	212
	3	4.71	0.64	197
	4	4.81	0.51	137
	5	4.61	0.80	171

Note: Cluster 1: Relaxing in Nature Package; Cluster 2: Backpacking and Hiking Package; Cluster 3: Viewing Nature and Hunting Package; Cluster 4: Picnicking Package; Cluster 5: Purely Fishing.

Satisfaction was measured on a 5-point scale ranging from 1 = very dissatisfied to 5 = very satisfied.

## 5 Discussion

The purpose of this study was to determine how recreation participation has changed over five years on the Monongahela National Forest following the world financial crisis in fiscal year 2009. Five market segments were identified based on visitor participation in 18 outdoor recreation activities. The potential effect of the global economic downturn does not appear to be ubiquitous across market segments.

### 5.1 Study objective 1: identify and describe the potential market winners and losers

*Market Winner--*There are several actions that tourism providers on the Monongahela National Forest can take during mega events like an economic downturn. The greatest growth potential involves promoting and attracting visitors that participate in cluster 1 activities: Relaxing in Nature Package (The package was 13.9 percent larger in cluster membership during fiscal year 2009). This market segment made up the largest percent of the tourist population in this study, traveled farther (especially during fiscal year 2009), spent more nights away from home (especially during fiscal year 2009), and were more satisfied when compared to some other segments that were examined. What makes this cluster unique are activities such as viewing nature, hiking, relaxing, viewing wildlife, driving for pleasure, nature centers, history, nature study and staying in resorts, cabins, or other accommodations. Many visitors do not have the time to participate in a combination of preferred activities on their own (Hsieh et al., 1992). Tourism providers can make it more convenient for visitors by offering activity packages for this target market, especially during an economic downturn. There is a lot of room for creativity when developing travel packages because this activity set has the largest number of activities among the clusters that were examined. Nature centers can serve as a hub for these activity packages given more than half of cluster 1 members visit them during their stay. Providing overnight accommodation such as resorts and cabins as part of the package may also be a valid option, especially considering the lengthier trips that are taken.

It is not surprising that Relaxing in Nature Package was more popular during the financial crisis. As mentioned earlier, the recession was an extremely stressful time for most employees. National forests may play a role in easing stress during a crisis through relaxing in nature, but other factors could also contribute to this finding as will be discussed in the limitations section.

*Market Losers--*There was a decline in cluster 2 visitors (Backpacking and Hiking Package) during the financial crisis, but they were more satisfied than clusters 3 and 5. This segment includes a package of activities including hiking, viewing wildlife, and backpacking. The average visitor in this market traveled much farther during fiscal year 2009, especially when compared to cluster 5. Regional marketing may work better for this segment during a financial crisis.

Cluster 5 visitors (Purely Fishing) also were less likely to visit the forest during the financial crisis. Nearly all visitors of this cluster participated in fishing. Older anglers were less likely to participate in fishing during fiscal year 2009. Cluster 5 visitors also drove shorter distances during the crisis and when compared to other clusters, therefore, regional marketing may be more effective during a strong economy.

### 5.2 Study objective 2: examine the variety of outdoor recreation activities within each market segment by comparing them with an activity package typology that is presented in the literature review

Questions about variety within an activity package emerged from this study. The concept of variety is somewhat different than the concept of behavioral involvement which was defined by Kim et al. (1997) as "the time and intensity of effort expended in a particular activity (Sievänen et al., 2018, p. 1). The authors define activity variety as the number of activities represented in an activity package, especially the number of activities from different package classes that were defined by Hendee et al. (1971).

Cluster 1 (Relaxing in Nature Package) clearly has the most variety of activities among all five clusters. In fact, it is the only cluster that falls under three different activity package classes (i.e., Appreciative-Symbolic, Passive-Free Play, and Sociable-Learning) as defined by Hendee et al. (1971). There are at least nine activities that make it different than most other clusters. As mentioned earlier, the variety of activities within this cluster can allow tourism providers to be more creative when designing a travel package with relaxing and viewing nature as core activities. Cluster 1 was also the most popular among visitors and received the highest trip satisfaction scores in 2014 and second highest satisfaction scores in 2009 further emphasizing the potential benefit of activity variety or choice in a travel package.

Cluster 2 (Backpacking and Hiking Package) had at least three activities that made it unique. Those activities tend to be characterized as Appreciative-Symbolic. Backpacking and hiking can be complementary during a forest visit (e.g., backpacking to a campsite can be followed by day hiking that allows visitors to explore an area). Perhaps less surprising, viewing wildlife appears to be an activity that accompanies backpacking and hiking. Providing wildlife viewing areas and interpretive signs along trails, where appropriate, can enhance this activity package.

Cluster 3 (Viewing Nature and Hunting Package) does not have a lot of variety because it only had two unique activities. However, those activities are opposites on the consumptive and non-consumptive dichotomy. Classifying this activity package as extractive would be an oversimplification. Hunters also appear to enjoy more non-consumptive activities like viewing nature. This has important implication for tourism providers. For example, hunting guides should provide information about a wider variety of nature during the hunting experience.

Cluster 4 (Picnicking Package) had at least four activities that made it unique and more diverse than clusters 3 and 5. The activities represent the Appreciative-Symbolic and Passive-Free Play classes. According to Hultsman et al. (1987), picnic areas are the most poorly planned recreation facility. One reason for the poor design is that the layout often fails to encourage other uses complimentary to picnicking. That is, picnic areas should be more than just picnic areas. This study suggests that biking and hiking trails should be near some picnic areas on the Monongahela National Forest to provide this opportunity.

Cluster 5 (Purely Fishing) only had one unique activity and was the least diverse cluster in the study. Fishing represents the Extractive-Symbolic class of the activity typology and suggest that not all outdoor recreation takes place in a package. (This may not be true for fishing opportunities offered on private land). Fishing parks, fishing events, and other focused fishing opportunities have the potential to be successful within the Monongahela National Forest. In addition, these findings support the notion that the variety of outdoor recreation activities within clusters may vary along a continuum from non-exclusive (cluster 1) to highly exclusive (cluster 5).

Cluster 3 and certainly cluster 5 are among the least diverse clusters in the study. It is interesting to note that both clusters also had the lowest overall satisfaction scores during fiscal years 2009 and 2014. It is possible that "activities in the same cluster provide similar satisfactions. Thus, for many people, some of those activities may be substitutable with little loss of satisfaction" (Hendee and Burdge, 1974, p. 106). Clusters 3 and 5 may offer little substitutability making it difficult to deal with the potential effects of crowding and displacement (Manning, 2011). Additionally, tourists may find it more difficult to substitute a more expensive activity with a less expensive one when fewer options are available.

Future research that utilizes NVUM data or data collected from forests or parks to segment visitors based on their activity participation could benefit from knowing five clusters were adequate when describing the range of markets in this study. This is generally consistent with the recent literature on activity-based market segmentation where four segments were the average. Comparing this study's findings with future research as it relates to forest recreation and tourism is needed and will fill a gap in the literature.

### 5.3 Study limitations

The main limitation of this study is the lack of causation that can be proven in a trend study. There may have been other factors that contributed to this study's results. However, the financial crisis was the dominant news event during fiscal year 2009 and the authors' data collection was well timed during that period. Future research is still needed to better understand the findings presented in this paper.

## 6 Conclusion

Five activity clusters describe the range of market segments on the Monongahela National Forest. That is, all 18 activities were significantly associated with cluster membership (*p* < .001), but the clusters often did not match the activity package typology. The most significant finding of this study was identifying the Relaxing in Nature Package (cluster 1) which was more popular during the financial crisis. Little, if any, research had documented positive impacts of the global economic downturn on tourism at the local level. However, by examining specific market segments at a smaller scale, the authors were able to identify potential opportunities for tourism providers during challenging times. Relaxing in Nature Package may provide the greatest opportunity for growth given this is the most popular activity package and it includes visitors that tend to spend more nights than some other clusters of visitors.

Many Monongahela National Forest visitors also traveled farther to get to the forest and stayed longer during fiscal year 2009, potentially making regional marketing more important during a financial crisis. This is somewhat different than what happened in parts of Europe (e.g., Germany) where tourists traveled closer, shorter, and cheaper (Reinhardt, 2011). This also contradicts Rittchainuwat et al. (2014) conclusions that tourists seek short-distance destinations during a financial crisis (at least in terms of driving distance). Although many visitors to the Monongahela National Forest traveled farther and stayed longer, their trips may have been cheaper than other options such as air transportation. Other factors such as fuel prices could have contributed to these findings.

No studies have been published that used fiscal year 2009 NVUM data to examine the potential impact of the financial crisis on visitation. Future research should take advantage of this data to see if the same market winners and losers are identified across the country. This research could also be compared with US Forest Service data that is scheduled to be collected around the time of the Coronavirus epidemic. COVID-19 is likely to be different than the 2008–2009 financial crisis because it also includes a medical crisis wherein demand is influenced by laws that are based on the emergency state and not just economic reasons. These similarities and differences should be explored, and activity-based segmentation may prove useful when making those comparisons. Tourism and crisis are a never-ending story. This study will help tourism providers on the Monongahela National Forest deal with current and future financial crises.

## Declarations

### Author contribution statement

Chad D. Pierskalla: Conceived and designed the experiments; Performed the experiments; Analyzed and interpreted the data; Contributed reagents, materials, analysis tools or data; Wrote the paper.

Connor B. Akers: Analyzed and interpreted the data; Contributed reagents, materials, analysis tools or data; Wrote the paper.

Jinyang Deng: Conceived and designed the experiments; Analyzed and interpreted the data; Wrote the paper.

David A. Smaldone: Analyzed and interpreted the data; Wrote the paper.

### Funding statement

Funding was provided by the 10.13039/100006959US Forest Service. USFS Agreement No: 09-PA-11092100-002.

### Data availability statement

Data will be made available on request.

### Declaration of interests statement

The authors declare no conflict of interest.

### Additional information

No additional information is available for this paper.
